# The Non-linear Relationship Between Normal Range Systolic Blood Pressure and Cardiovascular or All-Cause Mortality Among Elderly Population

**DOI:** 10.3389/fcvm.2021.677189

**Published:** 2021-07-27

**Authors:** Shuo Sun, Xiao-cong Liu, Guo-dong He, Kenneth Lo, Ying-qing Feng, Yu-qing Huang

**Affiliations:** ^1^Department of Cardiology, Guangdong Provincial People's Hospital, Guangdong Cardiovascular Institute, Guangdong Academy of Medical Sciences, Guangzhou, China; ^2^Department of Epidemiology, Centre for Global Cardio-Metabolic Health, Brown University, Providence, RI, United States; ^3^Department of Applied Biology and Chemical Technology, The Hong Kong Polytechnic University, Hong Kong, China

**Keywords:** normal blood pressure, systolic blood pressure, elderly population, cardiovascular mortality, all-cause mortality

## Abstract

**Purpose:** The aim was to explore the association of normal range SBP with cardiovascular and all-cause mortality in older adults without hypertension.

**Methods:** Participants aged ≥ 65 years without hypertension and those had an SBP level between 90 and 129 mmHg were included from the National Health and Nutrition Examination Survey (1999–2014). SBP was categorized into: 90–99, 100–109, 110–119, and 120–129 mmHg. Multivariate Cox regression was performed with hazard ratio (HR) and 95% confidence interval (CI).

**Results:** Of the 1,074 participants, 584 were men (54.38%). Compared with participants with SBP level ranged 110 to 119 mmHg, the HRs for all-cause mortality risk was 1.83 (95% CI: 1.04, 3.23) for SBP level ranged 90 to 99 mm Hg, 0.87 (95% CI: 0.54, 1.41) for SBP level ranged 100 to 109 mmHg, and 1.30 (95% CI: 0.96, 1.75) for SBP level ranged 120 to 129 mmHg (P for trend = 0.448), and the HR for cardiovascular mortality risk was 3.30 (95% CI: 0.87, 12.54) for SBP level ranged 90 to 99 mmHg, 0.35(95% CI: 0.08, 1.56) for SBP level ranged 100 to 109 mmHg, and 1.75 (95% CI: 0.78, 3.94) for SBP level ranged 120 to 129 mm Hg (P for trend = 0.349) after confounders were adjusted.

**Conclusion:** These were a nonlinear association of normal range SBP level with all-cause and cardiovascular death in older adults.

## Introduction

Hypertension is one of the most common chronic diseases and remains the leading cause of death in worldwide (1). Hypertension is generally defined as having a systolic blood pressure (SBP) ≥ 140 mmHg and/or a diastolic blood pressure (DBP) ≥ 90 mmHg, and prehypertension was a SBP of 120–139 mmHg and/or a DBP of 80-89 mmHg (2–5). In addition, prehypertension was further divided into low (120–129/80–84 mmHg) and high (130–139/80–89 mmHg) prehypertension, respectively (6). Several previous meta-analyses demonstrated the increased risk for cardiovascular diseases (CVD) in people with low-range prehypertension, and a higher risk for mortality despite adjusting for cardiovascular risk factors (7–10). Furthermore, a recent study showed an increment of the risk of incident CVD with increasing SBP levels in persons without hypertension nor other traditional atherosclerotic cardiovascular disease risk factors (11). However, previous studies were mainly conducted among young and middle-aged population, but the evidence for older adults aged ≥ 65 years is lacking. Importantly, in 2017, the definition of hypertension has been adjusted to 130/80 mm Hg with a SBP/DBP by the American College of Cardiology (ACC)/American Heart Association (AHA) Task Force on Clinical Practice Guidelines (12). These changes were largely driven by the increasing importance of hypertension control in preventing CVD (12, 13). Importantly, among elderly population free of hypertension, the association of 2017 ACC/AHA elevated hypertension (120–129/80mmHg) and normal blood pressure (<120/80 mmHg) with the risk of mortality were still unclear. To address the knowledge gap, the aim of the present study was to explore the association of SBP level with cardiovascular and all-cause mortality in elderly population without hypertension.

## Methods

### Study Population

**Figure 1:**
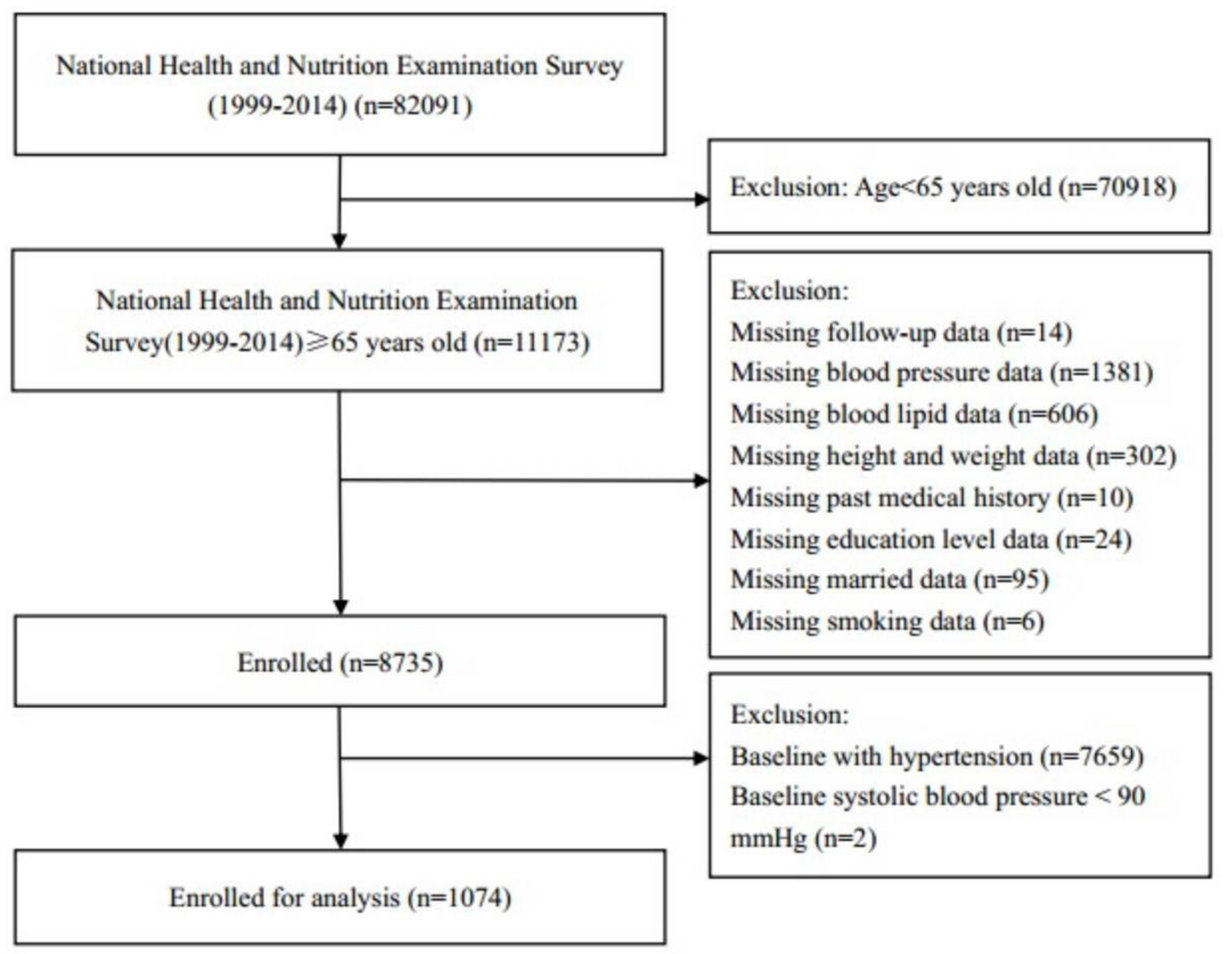
Research flow chart.

All participants were included from the 1999–2014 National Health and Nutrition Examination Surveys (NHANES). NHANES was an ongoing nationally representative study with a series of stratified, multistage probability surveys on United States civilian, non-institutionalized population, which was conducted by the National Center for Health Statistics of the Center for Disease Control and Prevention (14, 15). We enrolled subjects aged ≥ 65 years old. However, participants aged <65 years, with missing data on follow-up, blood pressure, blood lipid, height and weight, past medical history, education level, marital status and smoking status at baseline were excluded. In addition, participants with hypertension and SBP <90 mmHg were also excluded. Finally, a total of 1,074 participants were included for data analysis (Figure 1). The survey protocol was approved by the Institutional Review Board of the Centers for Disease Control and Prevention. All participants have provided written informed consent.

### Blood Pressure Measurement

Details of blood pressure measurement was described previously (15, 16). In brief, it was measured by a trained physician using a mercury sphygmomanometer [W. A. Baum Co. Inc (1050), Copiague, New York, USA] and an appropriately sized cuff. Three consecutive blood pressure readings were obtained from the same arm. SBP and diastolic blood pressure (DBP) were defined as the average value of three blood pressure measurements. Hypertension was defined as a previous diagnosis by a physician, and/or SBP/DPB ≥130/80 mmHg, and/or currently taking antihypertensive medications according to the 2017 ACC/AHA hypertension guideline (12). SBP level ranged from 90 to 129 mmHg was considered normal range. Participants were divided into four groups according to baseline SBP: 90–99, 100–109, 110–119, and 120–129 mmHg.

### Covariate Assessment

Data from questionnaires and physical examination were obtained according to a standardized procedure. Age, sex, race, marital status, smoking status, educational level, and history of comorbidities (including hypertension, diabetes, CVD and cancer) were self-reported during in-person interview. Medication history was obtained from self-report and the questions on prescribed medications. Other covariates included height, weight, total cholesterol, high-density lipoprotein cholesterol were also assessed. Body mass index (BMI) was defined as mass (kg) divided by the square of height (m^2^). Diabetes was defined as having a history of diabetes, or taking hypoglycemic medications currently, or fasting blood glucose level ≥ 7.0 mmol/l (126 mg/dl), or hemoglobin A1c (HbA1C) level ≥ 6.5% (17). Further details of data collection can be found in https://wwwn.cdc.gov/nchs/nhanes/Default.aspx.

### Outcomes

Outcomes of this study mainly were all-cause and cardiovascular mortality as obtained from a publicly available dataset of the NHANES. The database captured the vital status and cause of death of survey subjects from baseline to 31 December 2015 which came first (16). Cardiovascular mortality was defined according to the International Classification of Diseases, 10th Edition, Clinical Modification System codes (I00–I09, I11, I13, I20–I51, and I60–I69) derived from death-certificate data.

### Statistical Analysis

Baseline characteristics are presented as mean ± standard deviation (continuous variables) or percentage (categorical variables) as appropriate. We compared baseline characteristics among participants according to SBP level using Chi-square for categorical variables, and Analysis of Variance for continuous variables, respectively. Standardized Kaplan-Meier curves were used for survival analysis, and log-rank test was used to compare the differences in survival rate by SBP levels. The relationship between SBP levels and all-cause or cardiovascular mortality was examined by using Cox proportional hazards regression models, and hazard ratios (HRs) and 95% confidence interval (CI) were calculated. Model I only included SBP, and Model II was additionally adjusted for age, race, and sex. Model III was further adjusted for marital status, education level, smoking status, body mass index, DBP, total cholesterol, high density lipoprotein cholesterol, and pre-existing comorbidities (diabetes, cardiovascular disease, and cancer). Subgroup analysis were conducted according to body mass index (<25 or ≥ 25 kg/m^2^), sex (male and female), diabetes (yes and no), and race (White and non-White). Their interactions between diabetes and prehypertension status with all-cause and cardiovascular mortality were also tested. Given the inherent nature of multiple complex survey designs, we accounted for sample weight for each participant in the NHANES dataset. We used svydesign function in R to account for sampling weights, as well as the stratification and clustering. A 2-sided *P* < 0.05 was considered statistically significant. All statistical analyses were performed using R version 3.3.2 (R Foundation for Statistical Computing, Vienna, Austria).

## Results

### Baseline Characteristics

The baseline demographic characteristics were presented in Table 1. The study population included 1,074 subjects [584 (54.38%) male], average age was 72.20 ± 5.62 years. Participants with higher SBP level also had higher BMI, total cholesterol and DBP. However, there were no significant differences in age, sex, race, marital status, education level, smoking status, and comorbidities among SBP groups.

**Table 1:** Demographic and clinical characteristics according to normal systolic blood pressure levels.

		**Systolic blood pressure, mmHg**				
	**Total**	**90–99**	**100–109**	**110–119**	**120–129**	***P*-value**
Number	1074	41	162	401	470	
Age, years	72.20 ± 5.62	72.02 ± 5.33	71.86 ± 5.51	71.76 ± 5.49	72.71 ± 5.76	0.074
Sex, *n* (%)						0.774
Male	584 (54.38)	20 (48.78)	85 (52.47)	224 (55.86)	255 (54.26)	
Female	490 (45.62)	21 (51.22)	77 (47.53)	177 (44.14)	215 (45.74)	
Race, *n* (%)						0.593
Non-white	396 (36.87)	11 (26.83)	60 (37.04)	148 (36.91)	177 (37.66)	
White	678 (63.13)	30 (73.17)	102 (62.96)	253 (63.09)	293 (62.34)	
Marital status, *n*(%)						0.813
Married	415 (38.64)	16 (39.02)	68 (41.98)	151 (37.66)	180 (38.30)	
Other	659 (61.36)	25 (60.98)	94 (58.02)	250 (62.34)	290 (61.70)	
Education level, *n* (%)						0.226
Less than high school	346 (32.22)	11 (26.83)	44 (27.16)	126 (31.42)	165 (35.11)	
High school or above	728 (67.78)	30 (73.17)	118 (72.84)	275 (68.58)	305 (64.89)	
Smoking, *n* (%)						0.595
No	487 (45.34)	19 (46.34)	76 (46.91)	171 (42.64)	221 (47.02)	
Yes	587 (54.66)	22 (53.66)	86 (53.09)	230 (57.36)	249 (52.98)	
Body mass index, kg/m^2^	26.40 ± 4.79	25.03 ± 5.22	25.69 ± 5.06	26.58 ± 4.75	26.61 ± 4.66	0.038
Systolic blood pressure, mmHg	117.18 ± 8.47	95.86 ± 2.50	105.84 ± 2.79	115.07 ± 2.94	124.74 ± 2.92	<0.001
Diastolic blood pressure, mmHg	63.21 ± 11.53	53.64 ± 13.65	60.17 ± 12.01	63.31 ± 10.66	65.02 ± 11.28	<0.001
Total cholesterol, mg/dl	203.68 ± 39.41	215.44 ± 48.47	198.57 ± 38.88	201.32 ± 36.87	206.43 ± 40.52	0.018
HDL cholesterol, mg/dl	56.17 ± 16.49	62.98 ± 19.84	58.03 ± 14.96	54.81 ± 16.01	56.09 ± 16.93	0.008
**Comorbidities**, ***n*****(%)**						
Diabetes						0.293
No	913 (85.01)	37 (90.24)	140 (86.42)	347 (86.53)	389 (82.77)	
Yes	161 (14.99)	4 (9.76)	22 (13.58)	54 (13.47)	81 (17.23)	
Cardiovascular disease						0.835
No	969 (90.22)	37 (90.24)	143 (88.27)	364 (90.77)	425 (90.43)	
Yes	105 (9.78)	4 (9.76)	19 (11.73)	37 (9.23)	45 (9.57)	
Cancer						0.521
No	839 (78.12)	29 (70.73)	123 (75.93)	319 (79.55)	368 (78.30)	
Yes	235 (21.88)	12 (29.27)	39 (24.07)	82 (20.45)	102 (21.70)	
**Outcomes**, ***n*****(%)**						
Cardiovascular disease mortality						0.158
No	1027 (95.62)	38 (92.68)	159 (98.15)	386 (96.26)	444 (94.47)	
Yes	47 (4.38)	3 (7.32)	3 (1.85)	15 (3.74)	26 (5.53)	
All-cause mortality						<0.001
No	794 (73.93)	25 (60.98)	131 (80.86)	313 (78.05)	325 (69.15)	
Yes	280 (26.07)	16 (39.02)	31 (19.14)	88 (21.95)	145 (30.85)	

*n, number; HDL, high density lipoprotein.*

*Values are mean ± standardized differences or n (%)*.

### The Relationship Between Systolic Blood Pressure and Mortality

**Figure 2:**
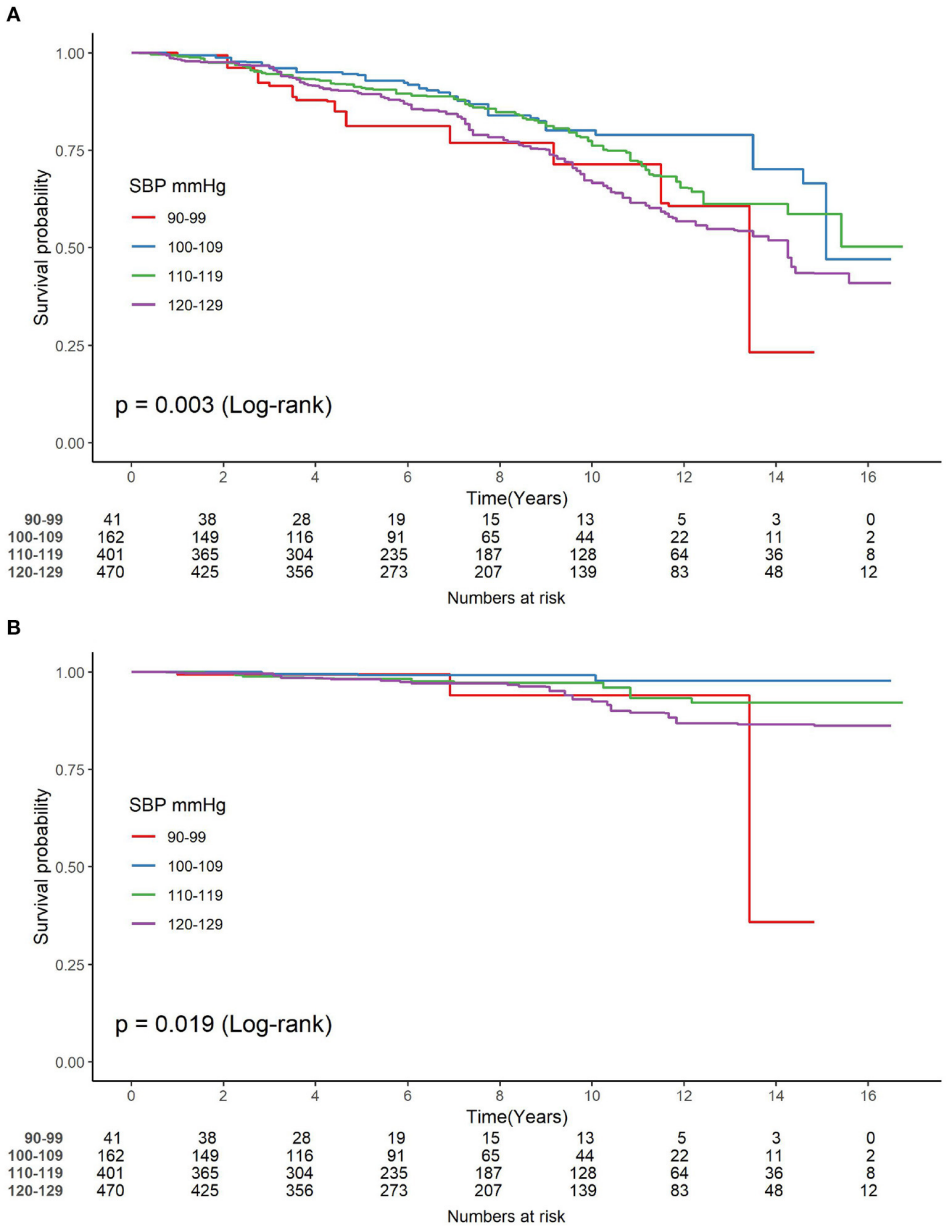
**(A,B)** Kaplan-Meier analysis for the incidence of mortality among groups of different normal systolic blood pressure level. SBP, systolic blood pressure.

During a median follow-up of 89.41 months, 47 (4.38%) cases of cardiovascular and 280 (26.07%) cases of all-cause mortality were observed, respectively. In addition, among all the 1,074 participants, there were 16 (39.02%), 31 (19.14%), 88 (21.95%) and 145 (30.85%) cases of all-cause mortality occurred ranging from 90–99 mmHg for SBP, 100–109 mmHg, 110–119 mmHg to 120–129 mmHg (*P* < 0.001), and 3 (7.32%), 3 (1.85%), 15 (3.74%), 26 (5.53%) cases of cardiovascular mortality occurred, respectively, among the above four groups (*P* = 0.158). Figure 2 showed the Kaplan-Meier mortality rate by the groups according to SBP level. The log-rank test revealed that there was a significant difference among each group of SBP in all-cause mortality (Figure 2A) and cardiovascular mortality (Figure 2B).

**Figure 3:**
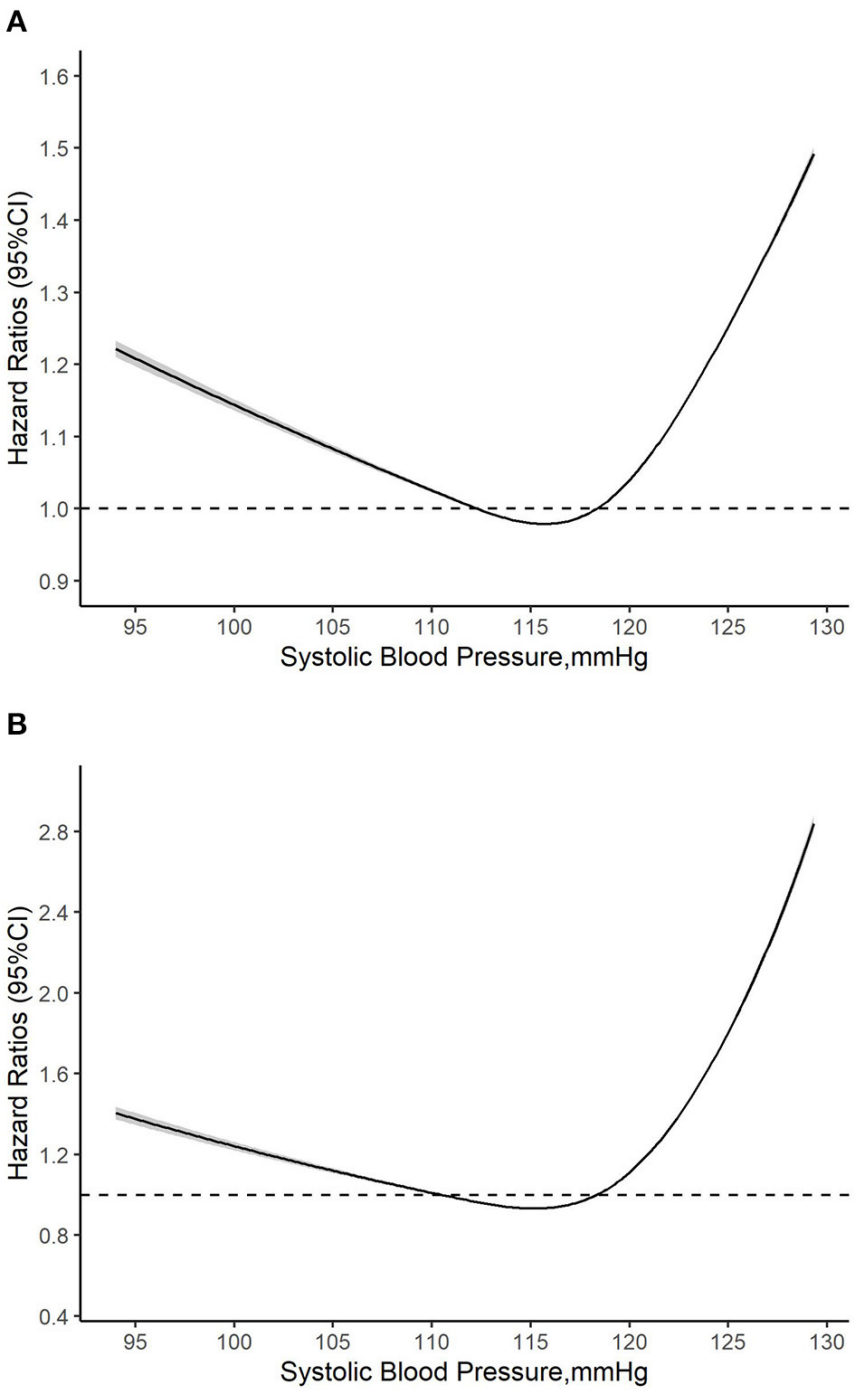
**(A,B)** Multivariate adjusted restricted cubic curve for the relationship between normal systolic blood pressure and mortality. HR, hazard ratio; CI, confidence interval. Age, sex, race, marital status, education level, smoking, body mass index, diastolic blood pressure, total cholesterol, high density lipoprotein cholesterol, and comorbidities (diabetes, cardiovascular disease, and cancer) were all adjusted.

As shown in Figure 3, the multivariate restrictive cubic curves showed that SBP has a non-linear relationship with all-cause (Figure 3A) and cardiovascular (Figure 3B) mortality, respectively. In addition, the risk of mortality may be the lowest when the SBP range from 110 to 119 mmHg. As shown in Table 2, when SBP was treated as a continuous variable, SBP has no obvious relationship with all-cause and cardiovascular mortality regardless of confounder adjustments (all *P* > 0.05). However, when SBP was referred as a categorical variable, compared with participants with an SBP level of 110 to 119 mmHg, there seemed to be a significantly higher risk for all-cause mortality among participants with an SBP level of 90 to 99 mmHg (HR, 1.56; 95% CI, 0.86, 2.82) and 120 to 129 mmHg (HR, 1.36; 95% CI, 1.03, 1.80) (*P* for trend = 0.113) in Model I. In Model III where age, sex, race, marital status, education level, smoking, body mass index, DBP, total cholesterol, high density lipoprotein cholesterol, and comorbidities (diabetes, CVD, and cancer) were adjusted, similar increment of all-cause mortality risk was observed among participants with an SBP level of 90 to 99 mmHg (HR, 1.83; 95% CI, 1.04, 3.23; *P* = 0.037) and 120 to 129 mmHg (HR, 1.30; 95% CI, 0.96, 1.75; *P* = 0.086) (*P* for trend = 0.448). As for cardiovascular mortality, all-cause mortality risk was also seemed to be higher among participants with an SBP level of 90 to 99 mmHg (HR, 3.30; 95% CI, 0.87, 12.54; *P* = 0.080) and 120 to 129 mmHg (HR, 1.75; 95% CI, 0.78, 3.94; *P* = 0.176), but the association did not reach statistical significance.

**Table 2:** Multivariate Cox regression analysis of normal systolic blood pressure with mortality.

	**Number**	**Model I** **HR (95%CI), *P*-value**	**Model II** **HR (95%CI), *P*-value**	**Model III** **HR (95%CI), *P*-value**
**All-cause mortality**				
SBP (per 10 mmHg increment)	1074	1.16(0.98,1.38)0.085	1.06(0.87,1.29)0.576	1.09(0.89,1.35)0.402
Each 10 mmHg increase in participants with SBP <115 mmHg	410	1.02(0.68,1.54)0.923	0.91(0.62,1.35)0.645	1.02(0.67,1.57)0.923
Each 10 mmHg increase in participants with SBP ≥ 115 mmHg	664	1.60(1.18,2.16)0.002	1.58(1.15,2.19)0.005	1.61(1.14,2.29)0.008
**SBP group, mmHg**				
90–99	41	1.56(0.86,2.82)0.145	2.01(1.10,3.64)0.022	1.83(1.04,3.23)0.037
100–109	162	0.81(0.50,1.29)0.367	0.89(0.56,1.43)0.636	0.87(0.54,1.41)0.581
110–119	401	1.0	1.0	1.0
120–129	470	1.36(1.03,1.8)0.031	1.27(0.96,1.68)0.089	1.30(0.96,1.75)0.086
P for trend		0.113	0.592	0.448
**Cardiovascular mortality**				
SBP (per 10 mmHg increment)	1074	1.09(0.88,1.34)0.449	1.25(0.71,2.23)0.441	1.36(0.79,2.35)0.269
Each 10 mmHg increase in participants with SBP <115 mmHg	378	0.78(0.22,2.76)0.703	0.58(0.20,1.7)0.324	0.70(0.25,1.97)0.499
Each 10 mmHg increase in participants with SBP ≥ 115 mmHg	696	2.31(1.02,5.25)0.045	2.36(1.06,5.27)0.036	2.71(1.07,6.88)0.036
**SBP group, mmHg**				
90–99	41	2.54(0.64,10.05)0.185	3.18(0.78,13.02)0.107	3.30(0.87,12.54)0.080
100–109	162	0.35(0.08,1.48)0.152	0.38(0.09,1.64)0.194	0.35(0.08,1.56)0.170
110–119	401	1.0	1.0	1.0
120–129	470	1.58(0.69,3.59)0.277	1.57(0.73,3.36)0.245	1.75(0.78,3.94)0.176
P for trend		0.418	0.544	0.349

*SBP, systolic blood pressure; HR, hazard ratio; CI, confidence interval.*

*Model I adjust for none.*

*Model II adjust for age, sex, and race.*

*Model III adjust for age, sex, race, marital status, education level, smoking, body mass index, diastolic blood pressure, total cholesterol, high density lipoprotein cholesterol, and comorbidities (diabetes, cardiovascular disease, and cancer)*.

### Subgroup Analysis

The result of subgroup analysis was shown in Table 3. Compared to the reference group (SBP: 110–119 mmHg), participants with the level of SBP 90–99 mmHg had a higher risk of all-cause mortality among female population compared to male population (HR: 3.01 vs. 1.58), non-White population compared to White population (HR: 3.08 vs. 1.89), for people with BMI ≥ 25 compared to BMI <25 kg/m^2^ (HR: 3.12 vs. 1.09) and those without diabetes compared to those with diabetes (HR: 2.23 vs. 0.67). Similar results were also found in participants with the level of SBP was 120–129 mmHg. When the level of SBP was 90–99 mmHg, and compared to the reference group, we only found the risk for cardiovascular mortality might be higher in women, White population, without diabetes and people with BMI <25 kg/m^2^ (all *P* < 0.05). However, when the level of SBP was 120–129 mmHg, and compared to the reference group, we only found the risk for cardiovascular mortality might be higher in people with BMI ≥ 25 kg/m^2^ (HR, 2.93; 95% CI, 1.19, 7.21; *P* = 0.019). In addition, we found that only BMI interacted significantly with the association between SBP and cardiovascular mortality (P for interaction = 0.012), while there were no interaction between sex, race, diabetes status and cardiovascular and all-cause mortality (all *P*-interaction > 0.05).

**Table 3:** Subgroups analyses of normal systolic blood pressure with mortality.

		**Systolic blood pressure, mmHg**				
		**Hazard ratios (95%CI)**, ***P*****-value**				
**Characteristic**	**Number**	**90–99**	**100–109**	**110–119**	**120–129**	***P*-interaction**
**All-cause mortality**						
Sex						0.578
Male	584	1.58(0.76,3.27)0.219	0.65(0.38,1.10)0.108	1.0	1.01(0.69,1.47)0.972	
Female	490	3.01(1.31,6.94)0.010	1.45(0.56,3.75)0.446	1.0	1.88(0.98,3.62)0.059	
Race						0.721
Non-white	396	3.08(0.78,12.07)0.107	0.97(0.40,2.35)0.943	1.0	1.90(0.96,3.76)0.066	
White	678	1.89(1.03,3.45)0.039	0.91(0.53,1.56)0.732	1.0	1.27(0.91,1.77)0.157	
Body mass index, kg/m^2^						0.560
<25	456	1.09(0.49,2.41)0.837	0.62(0.31,1.21)0.161	1.0	1.14(0.74,1.77)0.558	
≥25	618	3.12(1.46,6.68)0.003	1.14(0.53,2.45)0.744	1.0	1.52(1.01,2.28)0.046	
Diabetes						0.082
No	913	2.23(1.22,4.08)0.009	0.84(0.46,1.52)0.564	1.0	1.45(1.04,2.03)0.029	
Yes	161	0.67(0.11,4.15)0.669	1.10(0.4,2.97)0.857	1.0	0.86(0.31,2.41)0.771	
**Cardiovascular mortality**						
Sex						0.605
Male	584	1.49(0.19,11.89)0.709	0.37(0.07,1.99)0.248	1.0	1.59(0.58,4.34)0.364	
Female	490	18.7(2.16,161.92)0.008	0.38(0.04,3.22)0.372	1.0	2.37(0.54,10.28)0.251	
Race						0.731
Non-white	396	0.00 (0.00, Inf) 0.999	0.67(0.05,8.24)0.754	1.0	0.92(0.22,3.77)0.909	
White	678	4.81(1.05,22.09)0.043	0.47(0.09,2.53)0.381	1.0	2.52(0.96,6.58)0.059	
Body mass index, kg/m^2^						0.012
<25	456	7.40(1.36,40.14)0.02	0.95(0.11,8.1)0.964	1.0	1.21(0.32,4.49)0.778	
≥25	618	1.15(0.11,12.33)0.908	0.00 (0.00, Inf) 0.997	1.0	2.93(1.19,7.21)0.019	
Diabetes						0.662
No	913	6.31(1.56,25.5)0.01	0.57(0.11,2.96)0.506	1.0	2.25(0.93,5.44)0.073	
Yes	161	0.66(0.04,11.12)0.774	0.00 (0.00, Inf) 0.999	1.0	3.04(0.59,15.57)0.182	

*CI, confidence interval.*

*When analyzing a subgroup variable, age, sex, race, marital status, education level, smoking, body mass index, diastolic blood pressure, total cholesterol, high density lipoprotein cholesterol, and comorbidities (diabetes, cardiovascular disease, and cancer) were all adjusted except the variable itself*.

## Discussion

The main findings from the present study of older individuals with normal blood pressure were (1) when SBP <115 mmHg, as SBP decreased, the risk of mortality gradually increased, and when SBP ≥115 mmHg the risk of mortality gradually increased with SBP level. The appropriate SBP level is probably 110–120 mmHg. (2) The risk for cardiovascular mortality was increased at a SBP ≥ 115 mmHg. (3) Although SBP was in the normal range, relatively higher or lower SBP levels have a higher risk of mortality in women and population with overweight or obesity. (4) The relationship between normal SBP and all-cause mortality was differed by sex, race, BMI, and the history of diabetes. (5) The SBP level in the normal range might have dose-response relationship with all-cause and cardiovascular mortality.

Our findings were consistent with a prior meta-analysis of individual data for one million adults in 61 prospective studies, which demonstrated that usual SBP ≥115 mm Hg might significantly elevate the risk for all-cause and cardiovascular mortality (18). However, some studies have found that when SBP (120–139 mmHg) did not significantly increase the risk of death among elder population (19–21). Although we found that SBP >115 mmHg in the elderly might increase the mortality risk for older adults, it was still unclear whether blood pressure treatment should be initiated earlier. Currently, a large number of hypertension guidelines recommend pharmacological treatment to be initiated when SBP/DBP ≥140/90 mm Hg in population with aged ≥ 65 years, and if tolerable, the SBP can be reduced to <130 mm Hg (1, 3, 4). The post-analysis of the Felodipine Event Reduction (FEVER) trial found that when the average blood pressure level after treatment was lower than 120/70 mmHg, the risk of stroke, cardiac events and total death were the lowest (22). SBP intervention trial (SPRINT) also demonstrated that targeting a SBP of <120 mmHg compared to <140 mmHg could significantly result in the lower rates of fatal and non-fatal major cardiovascular events and all-cause mortality (23).However, for patients with type 2 diabetes, targeting a SBP of <120 mm Hg, as compared with <140 mm Hg, did not reduce the rate of a composite outcome of fatal and non-fatal major cardiovascular events (24). Therefore, more studies on blood pressure management among elderly may be needed in the future, which may help to refine the SBP target (<120 mmHg) for older adults in line with the results from SPRINT, JATOS, VALISH trials. Besides, our finding might suggest a lower blood pressure threshold to define hypertension in elderly people.

In addition, subgroup analysis showed that the relationship between normal SBP and all-cause mortality was differed by sex, race, overweight/obesity, and diabetes. A previous meta-analysis also showed a sex difference in the relationship between SBP and death in elder population (18). Among ambulatory adults aged 75 years or older, treating to an SBP target of <120 mm Hg compared with an SBP target of <140 mm Hg has resulted in significantly lower rates of fatal and non-fatal major cardiovascular events and death from any cause (25). We found that SBP of 120–129 mm Hg might increase risk for all-cause mortality among subjects without diabetes, but SBP <120 mm Hg did not, and this observation was similar to previous studies (24, 26). We also found that older adults with BMI ≥ 25 kg/m^2^ with a SBP of 120–129 mm Hg significantly increased the risk of all-cause and cardiovascular mortality. For the elderly with overweight or obesity, they might benefit more by having SBP <120 mm Hg.

To cautiously interpret our findings, some limitations of the present study should be noted. First, the small sample size might limit the generalizability of findings. Second, SBP was only measured once at baseline. Third, multiple covariates self-reported, therefore recall bias was possible. Fourth, this study did not fully consider the residual confounding effects, for example, atherosclerotic cardiovascular disease score and physical activity and diet. Fifth, we only explored the association of SBP with mortality, but not with adverse CVD events. Sixth, in this study, very few participants aged ≥g80 years, and there was no relevant data on frail, disability indices, and dementia/cognitive decline. Another limitation is that the cardiovascular event rate was low in a long follow-up, which is possible that the participants are normotensive at the beginning of study, therefore they have a lower risk of CVD over the years. Despite this issue, the direction of association between SBP and CVD mortality agrees with our overall findings. Besides, the present study had several strengths. On the one hand, NHANES have a rigorous and standardized study protocol, and have an extensive quality control procedure in data collection. On the other hand, the long period of follow up and the inclusion of multiple ethnic groups made this result reliable. Besides, this is one of the few studies to explore the relationship between SBP and all-cause and cardiovascular mortality in elderly normotensive subjects over such a long-term, prospective follow-up.

## Conclusions

In conclusion, SBP might have a dose-response relationship with all-cause and cardiovascular mortality in older normotensive population. Despite having normal range of SBP, low SBP (90 to 99 mmHg) or elevated SBP (120 to 129 mmHg) might increase the risk of all-cause mortality for older adults. The blood pressure management of the elderly population should be individualized, and more attention needed to be paid to the elderly individuals without hypertension.

## Data Availability Statement

Publicly available datasets were analyzed in this study. This data can be found here: https://www.cdc.gov/nchs/nhanes/index.htm.

## Ethics Statement

The studies involving human participants were reviewed and approved by the Institutional Review Board of the Centers for Disease Control and Prevention. The patients/participants provided their written informed consent to participate in this study.

## Author Contributions

All authors made substantial contributions to the conception and design, acquisition of data, or analysis and interpretation of data, took part in drafting the article or revising it critically for important intellectual content, agreed on the journal to which the article will be submitted, gave final approval of the version to be published, and agree to be accountable for all aspects of the work.

## Conflict of Interest

The authors declare that the research was conducted in the absence of any commercial or financial relationships that could be construed as a potential conflict of interest.

## Publisher's Note

All claims expressed in this article are solely those of the authors and do not necessarily represent those of their affiliated organizations, or those of the publisher, the editors and the reviewers. Any product that may be evaluated in this article, or claim that may be made by its manufacturer, is not guaranteed or endorsed by the publisher.
